# Baseline Exposure to Antipsychotic Medication in Young People at Clinical High Risk for Psychosis: A 2‐Year Italian Follow‐Up Study

**DOI:** 10.1002/hup.70003

**Published:** 2025-02-17

**Authors:** Lorenzo Pelizza, Alessandro Di Lisi, Emanuela Leuci, Emanuela Quattrone, Derna Palmisano, Simona Pupo, Pietro Pellegrini, Marco Menchetti

**Affiliations:** ^1^ Department of Biomedical and Neuromotor Sciences Alma Mater Studiorum – Università di Bologna Bologna Italy; ^2^ Department of Mental Health and Pathological Addiction Azienda USL di Parma Parma Italy; ^3^ Pain Therapy Service Department of Medicine and Surgery Azienda Ospedaliero‐Universitaria di Parma Parma Italy

**Keywords:** antipsychotic, clinical high risk, follow‐up, outcome, psychosis, treatment, ultra high risk

## Abstract

**Objective:**

Despite various models examining baseline factors, predicting outcomes in individuals at clinical high risk for psychosis (CHR‐P) remains challenging. Specifically, neglecting factors like ongoing antipsychotic (AP) medications introduce bias and reduce method precision. The main aim of this research was to determine if the presence of AP prescription at baseline identifies a CHR‐P subgroup with worse prognostic outcomes over a 2‐year period.

**Methods:**

A group of 180 FEP individuals (92 CHR‐P/AP+, 88 CHR‐P/AP−) were evaluated at baseline and after 24 months using the PANSS and GAF scales. Individuals with baseline AP prescription were included in the CHR‐P/AP+ subgroup; those not taking APs were grouped as CHR‐P/AP−. Univariate Cox regression analysis and mixed‐design ANOVA were performed.

**Results:**

After 2 years, CHR‐P/AP+ had a higher rate of new hospitalization but lower rate of service disengagement. No significant inter‐group difference in psychosis transition rate was found. A “time‐×‐group” interaction effect on longitudinal improvement in PANSS total score was observed in CHR‐P/AP+ subjects.

**Conclusions:**

It is advisable to conduct a more extensive outcome evaluation beyond the psychometric criteria for CHR‐P and the mere consideration of psychosis transition. Such an approach would facilitate the identification of specific CHR‐P subgroups with divergent prognoses and different AP response.

## 1 Introduction

In 1996, McGorry and his team pioneered the concept of delivering preventive interventions in psychiatry through an innovative approach focusing on early detection/intervention for young people with psychosis (A. R. Yung et al. 1996). The authors developed a clinical staging model with a specific focus on “Clinical High Risk for Psychosis” (CHR‐P) mental states that reshaped the landscape of psychiatric practice. However, predicting the prognosis of individuals at CHR‐P remains a substantial clinical challenge despite several models focused on baseline variables (Sanfelici et al. 2020). Indeed, bias from deficient consideration of certain factors like ongoing antipsychotic (AP) treatment could harm the method precision (Raballo, Poletti, and Preti 2021).

In this respect, recent meta‐analysis reported that CHR‐P people with baseline AP exposure had higher incidence rates of psychosis transition compared to CHR‐P subjects without AP prescription across different follow‐ups (1–2 years) and independently from age, gender ratio, overall sample size, sample enrichment, and quality of the studies (Raballo et al. 2020a, 2023). In our prior 1‐year follow‐up investigation confirming a higher transition rate to psychosis, we also reported other interesting statistically significant differences between CHR‐P individuals with or without baseline AP exposure in terms of age (Di Lisi et al. 2024), clinical severity at entry, and short‐term outcomes (Pelizza et al. 2023a). Specifically, CHR‐P people with baseline AP prescription had older age and more severe levels of psychopathology at presentation (e.g., greater socio‐occupational functioning decline, higher scores in both positive, disorganized, and negative symptoms) (Zeng et al. 2022), as well as poorer prognostic indicators (e.g., higher 1‐year incidence rates of new hospitalization and urgent/non‐planned visit) (Pelizza et al. 2024; Raballo, Poletti, and Preti 2024; Poletti et al. 2024). Moreover, early use of AP was not associated with a positive effect on cognitive function in CHR‐P adolescents, while the absence of AP treatment was associated with better cognitive recovery (Zhang et al. 2024). This seems to suggest that AP exposure might not be the preferred choice for cognitive recovery in CHR‐P subjects, especially in adolescence.

Building upon these results, the primary *objective* of this extended research was to examine whether the presence of ongoing AP treatment at presentation could serve as an early indicator for identifying a CHR‐P subgroup that are likely to experience more adverse prognostic outcomes over a longer (*2‐year*) follow‐up period. Specifically, we assessed a broader range of clinically relevant outcomes beyond psychosis transition (too often the only prognostic parameter considered in the CHR‐P research) to more effectively distinguish AP‐naïve CHR‐P subjects at entry from those with AP medication exposure.

## 2 Methods

### 2.1 Setting and Participants

All participants were sequentially enrolled within the “Parma At‐Risk Mental States” (*PARMS*) program from January 2016 to December 2021. The program was diffusely implemented across all adolescent and adult mental health services in the Parma Department of Mental Health, (Northern Italy) (Pelizza et al. 2023b).

*Inclusion criteria* were (a) to seek specialized mental health assistance, (b) age 12–25 years, and (c) to meet CHR‐P criteria as defined by the CAARMS at the baseline assessment: that is, “Attenuated Psychotic Symptoms” (APS), “Brief Limited Intermittent Psychotic Symptoms” (BLIPS), and “Genetic Vulnerability” (GV) (A. R. Yung et al. 2005).

*Exclusion criteria* were: (a) history of prior overt affective or non‐affective psychotic episodes; (b) previous exposure to AP drug or current AP intake for a duration exceeding 4 weeks in the present illness episode; (c) known intellectual disability (IQ < 70); and (d) neurological or other medical disorder with psychiatric manifestations. Past use of AP medication (i.e., in previous illness episodes) was considered a proxy for potential past psychotic episodes, consistently with original CAARMS guidelines (A. R. Yung et al. 2005). A current AP prescription of < 4 weeks was required to minimize pharmacological interference with baseline assessment within the PARMS program (Pelizza et al. 2023a). Specifically, in accordance with the current official guidelines on the “Early Intervention in Psychosis” (EIP) (Schmidt et al. 2015; Orygen 2016; RER 2024), the initiation of AP medication at entry within the PARMS protocol had to be reserved when CHR‐P individuals (a) displayed rapid deterioration in daily functioning, (b) experienced a sudden escalation of psychotic symptoms, (c) exhibited an immediate risk of suicide or severe violence, or (d) failed to respond to any other psychosocial interventions.

Local ethical approvals were obtained for the research (Ethics Committee “Area Vasta Emilia Nord [CE‐AVEN] protocol n. 559/2020/OSS*/AUSLPR”). All procedures performed in this study involving human participants were in accordance with the ethical standards of the institutional and/or national research committee and with the 1964 Helsinki declaration and its later amendments. All participants (including minors and their parents) provided their written informed consent for their participation in the study.

### 2.2 Instruments

The psychopathological evaluation encompassed the CAARMS, the Health of the Nation Outcome Scale (HoNOS) (Wing et al. 1998), the Positive and Negative Syndrome Scale (PANSS) (Kay, Fiszbein, and Opler 1987), and the Global Assessment of Functioning (GAF) scale (APA 2013).

The *CAARMS* is a clinical interview designed to explore various aspects of attenuated psychopathology. Its “*Positive Symptoms*” subscale serves as the basis for defining both CHR‐P and psychosis criteria. CAARMS interviews were conducted by trained PARMS team members using the approved Italian version (CAARMS‐ITA) (Raballo et al. 2013). Regular CAARMS scoring workshops and supervision sessions were implemented to ensure excellent interrater reliability (Pelizza et al. 2019). Every 12 months in the follow‐up, CAARMS criteria were employed to identify a possible *psychosis transition*.

The *HoNOS* was developed to evaluate the health and social functioning of individuals with severe mental illness. As indicated by Gale and Boland (2019), subscale scores were derived by grouping items into four categories: (a) “Behavioral Problems,” (b) “Impairment,” (c) “Psychiatric Symptoms,” and (d) “Social Problems.” In this study, we used the Italian version of the HoNOS (Lora et al. 2001), which was previously administered in clinical populations with early psychosis (Leuci et al. 2022). We considered a score of ≤ 2 on the HoNOS items 9, 10, and 11 as index of *functional remission* (Kortrijk et al. 2012).

The *PANSS* is a widely used clinical interview developed for assessing psychopathology in psychosis. It was previously administered also in young people with early psychosis (Pelizza et al. 2020a; Poletti et al. 2022). As indicated by Shafer and Dazzi (2019), we considered five principal psychopathological dimensions: “Disorganization,” “Negative Symptoms,” “Positive Symptoms,” “Resistance,” and “Affect” (“Depression‐Anxiety”). We used the Italian version of the PANSS that was previously administered also in CHR‐P samples (Pelizza et al. 2020a). As index of *symptomatic remission*, we considered a score of ≤ 3 (corresponding to mild severity or less during the last month) on the 8 PANSS items indicated by the “Remission in Schizophrenia Working Group's criteria” (Andreasen et al. 2005).

We also assessed the presence and course of *persistent negative symptoms* as further relevant outcome parameter over time. In accordance with the more conservative clinical criteria for persistent negative symptoms in psychosis suggested by Buchanan (2007), the following three main characteristics were used in this investigation: (a) presence of at least moderate (i.e., score of ≥ 4 on the PANSS) for at least 3 negative symptoms or at least moderately severe (i.e., score of ≥ 5 on the PANSS) for at least 2 negative symptoms; (b) persistence of negative symptoms for at least 6 months and for an extended period of time prior to the study beginning (i.e., at least 4 weeks); and (c) absence of relevant levels of positive symptoms, depression and extrapyramidal symptoms as defined thresholds on accepted and validated rating scales (i.e., persistent score of ≤ 3 in all items included in the PANSS “Positive Symptoms” domain and in PANSS “Depression” and “Guilt Feelings” items, and concurrently in absence of extrapyramidal symptoms requiring anticholinergic treatment).

The *GAF* is a commonly used scale for evaluating clinical and socio‐occupational functioning in individuals with severe mental disorders. It had reliable psychometric properties in CHR‐P populations as well (Nkire et al. 2021; Poletti et al. 2021). In this investigation, we considered a current GAF score (during the last month) of > 60 at follow‐up as index of *functional remission* (Zhang et al. 2022).

Lastly, we completed a sociodemographic and clinical chart including data on gender, ethnic group, age at entry, and level of education (detailed information are available in Table S1). All instruments were administered both at baseline and every 12 months during the follow‐up period.

### 2.3 Procedures

The initial DSM‐5 diagnosis was established through assessments conducted by a minimum of two trained PARMS team members using the Structured Clinical Interview for DSM‐5 disorders (*SCID‐5*) (First et al. 2016). Moreover, following the *CAARMS* interviews, individuals identified as at CHR‐P and taking AP medications at entry were categorized in the *CHR‐P/AP+* subgroup. Those participants without AP prescription at presentation were grouped as *CHR‐P/AP−*.

Within 3 weeks from baseline assessment, all CHR‐P participants were assigned to a *multi‐disciplinary team* consisting of a clinical psychologist, an early rehabilitation case manager, and a psychiatrist. Specifically, as a first‐line psychopharmacological treatment, low‐dose atypical APs were administered (Poletti et al. 2020).

Information regarding medication (type and dosage) and outcome parameter rates (e.g., service disengagement, psychosis transition, new hospitalization) was collected both at baseline and throughout the 2‐year follow‐up period. Additional details can be found in Table S1. Moreover, “suicide attempt” was defined as potentially injurious, self‐inflicted behavior without a fatal outcome for which there was (implicit or explicit) evidence of intent to die (Silverman et al. 2007). Data specifically derived from direct information reported by the patient and/or a relative well informed about the facts, or were documented in the clinical notes. “Self‐harm behavior” was defined as acts of deliberate self‐harm or intoxication with alcohol or drugs, but where there was no clear intention to die (Silverman et al. 2007). “Current suicidal ideation” was defined as a score of > 2 on the item 4 (“Suicidality”) of the Brief Psychiatric Rating Scale (BPRS), corresponding at least to occasional suicidal thinking without specific plan (e.g., “she/he feels they would be better off dead”) (Pelizza et al. 2020b). Service disengagement was defined as a complete lack of contact or untraceable for at least 3 months despite a need of treatment, counted from the date of the last face‐to‐face meeting with the clinical staff (Robson and Greenwood 2022). Finally, “functional recovery” was also simply defined as the return to work/school (Silva and Restrepo 2019).

### 2.4 Statistical Analysis

The data analysis was conducted using the Statistical Package for Social Science (SPSS) version 15.0 for Windows (SPSS Inc. 2010). Categorical variables were presented as frequencies and percentages, while continuous parameters as mean values and standard deviations. All statistical tests were two‐tailed, with a significance level set at 0.05.

As for outcome parameters, after having previously checked that the proportionality‐of‐hazards assumption was met (Atem, Matsouaka, and Zimmern 2019), univariate models were fitted for each outcome parameters across the 2 years of follow‐up using *Cox regression analysis*. Finally, a *mixed‐design ANOVA model* was also performed to evaluate the temporal stability of HoNOS, PANSS, and GAF scores within and between the two CHR‐P subgroups along the follow‐up period (Ranganathan et al. 2017). In this respect, for the within‐subject effects, when the assumptions of normality and homogeneity of variance were violated, we specifically used the Greenhouse‐Geisser correction (Geisser and Greenhouse 1958) and the adjustments to the degrees of freedom (Hyunh and Feldt 1970) because such statistical methods may correct for issues that can arise should the sphericity of the covariance matrix assumption be violated (Howell 2010).

## 3 Results

A total of 180 individuals at CHR‐P were recruited (90 [50%] males, 159 [88.3%] white Caucasians, mean age = 19.53 ± 3.79 years) (see Table S1 for detailed information on sociodemographic and clinical characteristics of the two CHR‐P subgroups).

Within the total CHR‐P group, 140 participants fulfilled the APS criteria (77.7%), 30 the BLIPS criteria (16.66%), and 10 the GV criteria (5.55%). At baseline (T0), 92 (51.1%) CHR‐P subjects had an AP prescription (mean equivalent dose of chlorpromazine = 101.25 ± 141.75 mg/day) and were categorized in the CHR‐P/AP+ subgroup. The remaining 88 participants were included in the CHR‐P/AP− subgroup. Among the CHR‐P/AP+ participants, 40 (43.5%) were taking risperidone, 18 (19.6%) olanzapine, 12 (13.1%) aripiprazole, 9 quetiapine, and 9 other AP drugs.

Over the course of the study, 153 (85.0%) CHR‐P participants successfully concluded the 2‐year follow‐up period (T2). Among the remaining 22 subjects showing early service disengagement, 6 individuals left the PARMS program and actively declined further contact with the treatment staff or could not be located (5 of them in the first 12 months), 12 ceased treatments due to clinical improvement, and 4 relocated outside the catchment area, making it impossible to maintain contact for the follow‐up assessment (see Figure S1 for details).

### 3.1 Baseline

As described in our previous paper (Pelizza et al. 2023a) (see Table S2 for details), compared to CHR‐P/AP− at baseline, CHR‐P/AP+ individuals showed older age (20.83 ± 3.37 vs. 18.17 ± 3.73; *z* = −4.722; *p* = 0.0001) and a higher percentage of past hospitalization in psychiatric ward before entering the PARMS programs (28.8% vs. 8.0%; *χ* ^2^ = 7.573; *p* = 0.006). They also had a higher PANSS “Positive Symptoms” (11.40 ± 3.90 vs. 10.18 ± 3.59; *z* = −2.123; *p* = 0.045) and “Negative Symptoms” (19.79 ± 6.75 vs. 17.97 ± 7.43; *z* = 2.531; *p* = 0.048) subscores, as well as a lower GAF score (47.67 ± 7.16 vs. 49.83 ± 9.82; *z* = 2.198; *p* = 0.043). Finally, in comparison with AP‐naïve, CHR‐P/AP+ individuals had higher baseline prevalence rates of diagnosis of both BLIPS and brief psychotic disorder (Table 1).

**TABLE 1:** Baseline clinical comparisons between the two CHR‐P subgroups (*n* = 180).

Variable	CHR‐P/AP+ (*n* = 92)	CHR/AP− (*n* = 88)	*χ* ^2^/*z*	*p*
CHR‐P criteria				
BLIPS	23 (25.0%)	7 (8.0%)	9.409	**0.002**
APS	64 (69.6%)	76 (86.4%)	7.343	**0.007**
GV	5 (5.4%)	5 (5.6%)	0.275	0.600
Baseline DSM‐5 diagnosis				
Brief psychotic disorders	17 (18.5%)	7 (8.0%)	4.311	**0.038**
Psychotic disorder NOS	18 (19.6%)	11 (12.5%)	0.106	0.102
Schizotypal personality disorder	15 (16.3%)	11 (12.5%)	0.298	0.201
Borderline personality disorders	3 (3.3%)	10 (11.4%)	4.407	**0.036**
Obsessive‐compulsive disorders	4 (4.3%)	4 (4.5%)	0.097	0.957
Eating disorder	1 (1.1%)	3 (3.4%)	0.248	0.113
Depressive disorders	27 (29.3%)	35 (39.8%)	0.375	0.201
Anxiety disorder	7 (7.6%)	7 (8.0%)	0.099	0.974

*Note:* Frequencies (and percentages) and mean ± standard deviation are reported. Chi‐square (*χ* ^2^) test and Mann–Whitney *U* test (*z*) values are reported. Statistically significant *p* values are in bold.

Abbreviations: AP, antipsychotic medication; APS, attenuated psychotic symptoms; BLIPS, Brief Limited Intermittent Psychotic Symptoms; CHR‐P, Clinical High Risk for Psychosis; CHR‐P/AP−, CHR‐P individuals without baseline AP prescription; CHR‐P/AP+, CHR‐P individuals with baseline AP exposure; DSM‐5, diagnostic and statistical manual of mental disorders‐5th edition; GV, genetic vulnerability; NOS, not otherwise specified.

### 3.2 Follow‐Up

Across the follow‐up, CHR‐P/AP+ participants showed a higher 2‐year rate of new hospitalization compared to CHR‐P/AP− subjects (Table 2). Moreover, although there were no noteworthy between‐group differences regarding 2‐year incidence rates of psychosis transition, CHR‐P criteria persistence, suicide attempt, self‐harm behavior, suicidal ideation, GAF functional remission, HoNOS functional remission, PANSS symptomatic remission, and the presence of persistent negative symptoms, CHR‐P/AP+ participants showed a lower 2‐year incidence rate of *service disengagement* in comparison with AP‐naïve individuals.

**TABLE 2:** Univariate Cox proportional‐hazard models for 2‐year outcome parameters in the two CHR‐P subgroups (*n* = 180).

Variable	CHR‐P/AP+ (*n* = 92)	CHR‐P/AP− (*n* = 88)	Statistic test			
			HR	95% CI		*p*
				Lower	Higher	
2‐year psychosis transition	18 (19.6%)	11 (12.5%)	1.488	0.703	3.152	0.299
2‐year CHR‐P criteria persistence	34 (37.0%)	27 (30.7%)	1.080	0.651	1.789	0.767
2‐year new hospitalization	20 (21.7%)	6 (6.8%)	3.092	1.242	7.701	**0.015**
2‐year suicide attempt	6 (6.5%)	7 (8.0%)	0.763	0.256	2.271	0.627
2‐year self‐harm behavior	7 (7.6%)	13 (14.8%)	0.486	0.194	1.219	0.124
2‐year current suicidal ideation	29 (31.5%)	36 (40.9%)	0.684	0.419	1.116	0.128
2‐year service disengagement	9 (9.8%)	18 (20.5%)	0.512	0.239	1.133	**0.048**
2‐year functional recovery	56 (60.9%)	60 (68.2%)	0.796	0.553	1.145	0.219
2‐year GAF functional remission	55 (59.8%)	61 (69.3%)	0.772	0.536	1.111	0.163
2‐year HoNOS functional remission	77 (83.7%)	70 (79.5%)	0.939	0.679	1.298	0.702
2‐year PANSS symptomatic remission	75 (81.5%)	60 (68.2%)	1.070	0.762	1.502	0.697
2‐year persistent negative symptoms	12 (13.0%)	8 (9.1%)	1.279	0.523	3.129	0.590

*Note:* Significant statistical *p* values are in bold.

Abbreviations: 95% CI, 95% confidence intervals for HR; AP, antipsychotic medication; CHR‐P, Clinical High Risk; CHR‐P/AP−, CHR‐P individuals without baseline AP prescription (AP‐naive); CHR‐P/AP+, CHR‐P individuals with baseline AP prescription; GAF, Global Assessment of Functioning; HoNOS, Health of the Nation Outcome Scale; HR, Hazard Ratio; *p*, statistical significance; PANSS, Positive And Negative Syndrome Scale.

The results of the mixed‐design ANOVA on repeated measures (“within‐subject” effects) revealed a statistically significant impact of time on all clinical parameters (HoNOS, GAF, and PANSS scores) (Table 3). Although no significant group differences (“between‐subjects” effects) were found, there were statistically relevant “time x group” *interaction effects* in *PANSS* total score and in “Disorganization” and “Affect” factor subscores, as well as in *HoNOS* total score and in HoNOS “Impairment” domain subscore. Specifically, when considering the estimated marginal means and their corresponding *p*‐values (for details on profile plots over time, see also Figure S2), CHR‐P/AP+ participants showed greater longitudinal improvements in all these PANSS and HoNOS scores.

**TABLE 3:** Mixed‐design ANOVA results: Psychopathological and outcome parameters across the 2‐year follow‐up period in the two CHR‐P subgroups.

Variable	Time effect				Group effect				Interaction effect (time × group)			
	df	*F*	*p*	*η* ^2^	df	*F*	*p*	*η* ^2^	df	*F*	*p*	*η* ^2^
HoNOS behavioral problems	1.5	65.882	**0.0001**	0.285	1	1.137	0.288	0.007	1.5	1.282	0.274	0.008
HoNOS impairment	1.8	45.696	**0.0001**	0.217	1	0.562	0.455	0.003	1.8	5.668	**0.005**	0.033
HoNOS psychiatric symptoms	1.5	153.771	**0.0001**	0.482	1	0.008	0.927	0.001	1.5	2.473	0.103	0.015
HoNOS social problems	1.8	301.249	**0.0001**	0.361	1	0.953	0.330	0.006	1.8	2.507	0.090	0.015
HoNOS total score	1.5	183.775	**0.0001**	0.527	1	0.157	0.693	0.001	1.5	4.651	**0.018**	0.027
GAF	1.4	138.956	**0.0001**	0.565	1	0.456	0.501	0.003	1.4	1.414	0.244	0.013
PANSS positive	1.2	7.941	**0.003**	0.062	1	0.291	0.655	0.002	1.2	2.919	0.082	0.024
PANSS negative	1.5	60.731	**0.0001**	0.336	1	0.072	0.789	0.001	1.5	2.051	0.144	0.017
PANSS disorganization	1.5	15.110	**0.0001**	0.111	1	0.232	0.631	0.002	1.5	6.463	**0.005**	0.051
PANSS affect	1.3	67.121	**0.0001**	0.357	1	0.001	0.983	0.001	1.3	5.670	**0.012**	0.045
PANSS resistance/excitement	1.4	0.327	0.647	0.003	1	1.788	0.184	0.015	1.4	0.073	0.869	0.001
PANSS total score	1.4	47.572	**0.0001**	0.284	1	0.099	0.753	0.001	1.4	5.533	**0.011**	0.044

Variables	CHR‐P/AP+				CHR‐P/AP−			
	T0 EMM (SE)	T2 EMM (SE)	T0–T2 MD (SE)	*T* (*p*)	T0 EMM (SE)	T2 EMM (SE)	T0–T2 MD (SE)	*T* (*p*)
HoNOS impairment	2.398	1.170	1.187	7.560	1.848	1.241	0.738	4.097
	(0.197)	(0.144)	(0.157)	(**0.0001**)	(0.208)	(0.152)	(0.180)	(**0.0001**)
HoNOS total score	20.750	11.341	9.593	11.497	18.759	11.380	7.631	10.510
	(0.815)	(0.760)	(0.834)	(**0.0001**)	(0.860)	(0.802)	(0.726)	(**0.0001**)
PANSS disorganization	16.190	13.069	3.121	4.872	15.185	14.492	0.692	1.179
	(0.701)	(0.682)	(0.640)	(**0.0001**)	(0.663)	(0.664)	(0.587)	(0.243)
PANSS affect	16.069	11.379	4.690	6.800	14.785	12.308	2.477	5.255
	(0.680)	(0.637)	(0.690)	(**0.0001**)	(0.642)	(0.602)	(0.471)	(**0.0001**)
PANSS total score	72.807	55.965	16.842	6.269	68.796	60.708	8.062	3.817
	(2.297)	(2.802)	(2.687)	(**0.0001**)	(2.151)	(2.624)	(2.112)	(**0.0001**)

*Note:* As all Mauchly's tests of sphericity are statistically significant (*p* < 0.05), Greenhouse–Geisser corrected degrees of freedom to assess the significance of the corresponding *F* value are used. Statistically significant *p* values are in bold. Statistical trends in *p* value (*p* < 0.01) are underlined.

Abbreviations: *η* ^2^, partial eta squared; ANOVA, analysis of variance; AP, antipsychotic medication; CHR‐P, Clinical High Risk for Psychosis; CHR‐P/AP−, CHR‐P participants without AP prescription at entry; CHR‐P/AP+, CHR‐P participants with AP prescription at entry; df, degrees of freedom; EMM, estimated marginal mean; *F*, *F* statistic value; GAF, Global Assessment of Functioning; HoNOS, Health of the Nation Outcome Scale; MD, mean difference; *p*, statistical significance; PANSS, Positive And Negative Syndrome Scale; SE, standard error; *t*, Student's *t* statistical value; T0, baseline assessment; T2, 2‐year assessment time.

## 4 Discussion

Although not revised in the last 7 years, international guidelines generally advise against using AP medication as first‐line treatment in young people at CHR‐P. Instead, they recommend considering APs only when psychosocial interventions (particularly individual psychotherapy) have proven to be ineffective (Orygen 2016).

In this investigation, we observed a significantly *higher baseline prevalence rate of AP prescription* (51.1%) as compared to the 23.3% reported in a recent meta‐analysis (Raballo, Poletti, and Preti 2020b). In over half of our CHR‐P individuals at entry, the severity of their clinical condition (which extended beyond positive symptoms to include functioning decline and negative symptoms) prompted healthcare providers to consider AP prescription (see also our previous findings [Pelizza et al. 2023a] for further details). Specifically, compared to CHR‐P/AP−, our CHR‐P/AP+ showed higher baseline severity levels in both positive and negative symptoms, as well as lower baseline GAF scores (see Table S1). Therefore, as previously cited in the current literature, decisions by the clinicians why some individuals are prescribed AP medication (also in higher doses) appear to be mainly related to a greater perceived clinical severity and a greater functional disability at entry (Poletti et al. 2024; Di Lisi et al. 2024). Indeed, according to the current official EIP guidelines (Schmidt et al. 2015; Orygen 2016; RER 2024), AP medications at entry in the PARMS protocol had to be reserved when CHR‐P individuals displayed rapid deterioration in daily functioning, experienced a sudden escalation of psychotic symptoms, and exhibited an immediate risk of suicide or severe violence, in addition with their failing to respond to psychosocial interventions.

Moreover, these results strongly emphasize the discrepancy between real‐world prescription practices and official guideline recommendations, particularly in the treatment of those young CHR‐P individuals characterized by higher baseline levels of psychopathology and poorer daily functioning. In this investigation, the *higher severity of clinical presentation* in CHR‐P/AP+ participants was also supported by their higher baseline prevalence rates of previous hospitalization, BLIPS, and brief psychotic disorder diagnosis. These findings overall support that the general pattern of AP prescription should be consider as a clinical indicator of severity of the ongoing psychopathological process and should be introduced as relevant baseline factor in prediction/prognostic models for CHR‐P populations (Belvederi Murri et al. 2023).

In this respect, a crucial question is whether the CHR‐P/AP+ population can be in fact considered as “false positives” and whether their CHR status and trajectories can be “overshadowed” by AP prescription (A. Yung, Phillips, and McGorry 2004). Indeed, these participants might “look like” fulfilling CHR criteria, but instead presenting with “partially treated psychosis,” hence lying on a different stage of illness altogether and non‐comparable with “true positive” CHR individuals (McGorry et al. 2010). For this reason, should baseline AP prescription be always included among the “classic” exclusion criteria from CHR‐P clinics and research (even if < 4 weeks in the current illness episode)? In our opinion, a unique update of international guidelines on this topic is absolutely necessary. Moreover, future studies evaluating the “upstream” process of AP prescription before the admission to specialized EIP services are needed, especially to investigate prescription patterns that are not in line with current CHR guidelines as potential proxy for non‐specialists’ providers of care's understanding of the at‐risk construct. Indeed, our CHR‐P/AP+ subgroup manifesting greater severity of clinical presentation and higher rates of past hospital admission and brief psychotic disorder diagnosis can be the result of a different clinical population (in stage 2 [“first episode psychosis”] rather Ib [“CHR‐P”]) (McGorry et al. 2010). However, the findings of this investigation pointed out an excessive use of AP medication in our Italian CHR‐P population. International differences in prescribing practices may go beyond mere clinical considerations and also involve socio‐cultural factors that inevitably influence the clinician's decisions. In our opinion, it is possible that different cultural contexts differently establish the threshold for considering a subjective, attenuated experience as a psychiatric symptom, and therefore susceptible to psychopharmacological treatment.

Our CHR‐P/AP+ participants were also *older* in comparison with CHR‐P/AP− subjects. This further supports the idea that AP prescription is less commonly used in younger age, although it did not involve an insignificant portion (30.8%) of CHR‐P adolescents (aged < 18 years) treated in the PARMS program.

At the end of our *follow‐up*, no inter‐group difference in terms of rates of *transition to psychosis* was found. Within the CHR‐P/AP+ subgroup, psychosis transition incidence is not as significantly higher as observed after 1 year of intervention (Pelizza et al. 2023a). This does not confirm that in cases of more severe CHR‐P psychopathology and poorer functioning at entry, AP medication use prevents the onset of psychosis (as observed in the short‐term [i.e., in the first year of treatment]). Certainly, baseline AP exposure was an early risk indicator of *new hospitalization* throughout the entire follow‐up period. This suggests that CHR‐P/AP+ individuals show worst outcome results beyond psychosis transition even if they were taking AP medication (Yung et al. 2003; Zhang et al. 2020).

However, baseline AP prescription seems to identify a CHR‐P subgroup specifically characterized by a *higher 2‐year service engagement* within EIP services and a *more intense response to specialized EIP treatments* over time, especially in terms of longitudinal improvement in psychopathology and cognitive/somatic disabilities (i.e., PANSS and HoNOS scores). In this respect, in commenting these findings, one important confounder may be the compliance with AP drug. Indeed, subjects with greater clinical severity might have worse insight and willingness to receive treatment, as well higher risk of abandoning it. However, no inter‐group difference in terms of PANSS G12 “Lack of judgment/insight” item subscore was found at presentation (mean ± standard deviation = 2.40 ± 1.30 vs. 2.11 ± 1.49; *z* = −0.175; *p* = 0.861). Furthermore, some crucial questions still remain unanswered: could AP therapy have a temporally longer preventive wave (i.e., requiring 2 years of intervention) to decrease clinical severity and psychosis transition risk at the same level as AP‐naïve individuals? Are these preventive effects primarily due to AP treatment, associated psychosocial treatments, or both? Future studies specifically examining effectiveness of these different components of EIP multidisciplinary interventions are thus needed.

### 4.1 Limitations

A first limitation is due to our observational design. Indeed, clinical data were collected not for medication‐related research purposes and there were no specific data available on AP side effects and patient's adherence/compliance. Moreover, as our main research goal was related to baseline AP exposure, no longitudinal information on AP prescription was examined.

Second, while following the recommendations of international guidelines on AP prescription in CHR‐P populations, our CHR‐P/AP+ participants could be represent “false positives,” and their status and trajectories may be overshadowed by AP prescription.

Third, no statistical analysis on concomitant pharmacological and psychosocial treatments was performed. Moreover, this was a single‐center research and may not be considered representative of all current real‐world treatment patterns.

Fourth, although widely used also in CHR‐P populations (Poletti et al. 2022), the PANSS is usually administered as a measure of symptomatic remission for patients with psychotic disorder (i.e., all PANSS‐8 items scores 3 or lower) and could be not appropriate as a measure of symptomatic remission for CHR‐P individuals. Therefore, future research using the CAARMS to assess symptomatic remission in CHR samples may be helpful. However, there is no international consensus on specific symptomatic remission criteria based on the CAARMS scores.

Fifth, another weakness was that no specific assessment on insight into the illness was used. Indeed, it should be included as a possible confounder, together with compliance with AP medication. CHR‐P individuals with higher psychopathological and functional severity might have worse insight and willingness to receive treatment, so more often abandoning specialized intervention. Therefore, future research specifically evaluating clinical insight is needed.

Sixth, no data on substance use/abuse in the follow up were collected. Indeed, substance use/abuse is another possible confounder on predicting outcomes in CHR‐P populations, and should be more specific (e.g., cannabis vs. other). Therefore, future longitudinal investigations assessing this variable are absolutely needed. Moreover, the issue of sociodemographic and clinical heterogeneity in individuals both at CHR‐P and with first episode psychosis is also probably another reason of difficulties in predicting outcomes.

Finally, another limitation was related to the relatively small sample size of our CHR‐P subgroups, which may indirectly imply low incidence rates in outcome parameters here examined. Further investigations on larger CHR‐P populations are thus needed.

## 5 Conclusion

The results of this investigation support the role of AP prescription at entry as an *early “warning flag”* of the higher risk of new hospitalization in the first 2 years of treatment. However, in the *longer* time, it seems to identify a CHR‐P subgroup with some *positive outcomes*, such as a lower service disengagement risk and a more substantial improvement in psychopathology and cognitive/somatic disability in CHR‐P individuals treated within our EIP service.

To date, we are still unable to identify a subgroup of CHR‐P patients who may benefit from treatment with AP at baseline. In our view, more comprehensive assessment is warranted, going beyond CHR‐P psychometric criteria and psychosis transition, and taking into account the subjective experiences of CHR‐P individuals and their broader life context beyond just positive symptoms. This expanded evaluation should encompass factors such as socio‐occupational functioning, quality of life, coexisting psychiatric conditions, persistent negative symptoms, past traumatic experiences, family dynamics, community inclusion, social/personal recovery, and content shared on social networks. By adopting this approach, we could expand the range of outcome measures in CHR‐P research beyond merely tracking the transition to full‐blown psychosis. It also would enable to pinpoint distinct CHR‐P subgroups that could have potentially different prognosis and AP treatment response.

## Conflicts of Interest

The authors declare no conflicts of interest.

## Supporting information

Supporting Information S1

## Data Availability

The data that support the findings of this research are available on request from the corresponding author. The data are not publicly available due to privacy or ethical restrictions.
