# Supplementary material for: Baseline Exposure to Antipsychotic Medication in Young People at Clinical High Risk for Psychosis: A 2‐Year Italian Follow‐Up Study
Source: Hum Psychopharmacol. 2025 Feb 17;40(2):e70003. doi: 10.1002/hup.70003 (PMC11832455; doi:10.1002/hup.70003)
Supplement: Supplementary file 1 — Supporting Information S1 [file HUP-40-e70003-s001.docx]

Table S1 – Sociodemographic and clinical information collected in the present study.

| *Baseline sociodemographic data*  Gender, ethnic group, migrant status, age at entry, level of education (in years), civil status, and living status  *Baseline clinical data*  Source of referral to the PARMS program, DUI, family history of psychosis, past specialist contact, antipsychotic prescriptions, acceptance of psychosocial interventions’ proposal, previous hospitalization, past suicide attempt, current substance abuse, and current suicidal ideation.  *Longitudinal clinical data* (after the 2-year follow-up period)  New hospitalization, new suicide attempt, new self-harm behavior, current suicidal ideation, service disengagement, and functional recovery. |
| --- |

Note – PARMS = Parma At-Risk Mental States; DUI = Duration of Untreated Illness (defined as the time interval between the onset of psychiatric symptoms and the first pharmacological and/or psychological treatment) (Matsumoto et al., 2021). Suicide attempt = potentially injurious, self-inflicted behavior without a fatal outcome for which there was (implicit or explicit) evidence of intent to die (Silverman et al., 2007), derived from direct information reported by the patient (or by a relative well informed about the facts) or documented in the clinical notes (Silverman et al., 2007). Self-harm behavior = acts of deliberate self-harm or intoxication with alcohol or drugs, but where there was no clear intention to die. Current suicidal ideation = score of > 2 on item 4 (“Suicidality”) of the Brief Psychiatric Rating Scale (BPRS), corresponding at least to occasional suicidal thinking without specific plan (e.g., “she/he feels they would be better off dead”) (Pelizza et al., 2020). Service disengagement = complete lack of contact or untraceable for at least 3 months despite a need of treatment, counted from the date of the last face-to-face meeting with the clinical staff (Robson & Greenwood, 2022). Functional recovery = return to school or work (Silva & Restrepo, 2019).

+) Matsumoto, Y., Nakamae, T., Abe, Y., Watanabe, A., Narumoto, J. (2021). Duration of untreated illness of patients with obsessive-compulsive disorder in Japan. Early Intervention in Psychiatry, 15, 1644-1649. <https://doi.org/10.1111/eip.13105>.

+) Pelizza, L., Poletti, M., Azzali, S., Paterlini, F., Garlassi, S., Scazza, I., Chiri, L.R., Pupo, S., Pompili, M., Raballo, A. (2020). Suicide risk in young people at Ultra-High Risk (UHR) of psychosis: findings from a 2-year longitudinal study. Schizophrenia Research, 220, 98-105. <https://doi.org/10.1016/j.schres.2020.03.051>.

+) Robson, E., Greenwood, K. (2022). Rates and predictors of disengagement and strength of engagement for people with a first episode of psychosis using early intervention services: a systematic review of predictors and meta-analysis of disengagement rates. Schizophrenia Bulletin Open, 1, <https://doi.org/10.1093/schizbullopen/sgac012>.

+) Silva, M.A., Restrepo, D. (2019). Functional recovery in schizophrenia. Revista Colombiana de Psiquiatria (English Edition), 48, 252-260. <https://doi.org/10.1016/j.rcp.2017.08.004>.

+) Silverman, M.M., Berman, A.L., Sanddal, N.D, O’Carroll, P.W., Joiner, T.E. (2007). Rebuilding the tower of Babel: a revised nomenclature for the study of suicide and suicidal behaviors - part 2: suicide-related ideations, communications, and behaviors. Suicide and Life Threating Behavior, 37, 264-277. <https://doi.org/10.1521/suli.2007.37.3.264>.

Figure S1 – Baseline AP exposure rate in the CHR-P total sample (n = 180).

T0

180 CHR-P individuals

40 (43.5%) risperidone

18 (19.6%) olanzapine

12 (13.1%) aripiprazole

22 other AP medication

92 (51.1%) **CHR-P/AP+**

88 (48.9%) **CHR-P/AP-**

T1

*175* CHR-P individuals

*concluded* the 1-year follow-up period

*5* CHR-P individuals

*service disengagement*

*5* CHR-P individuals

*dropped-out* from the PARMS program

91 CHR-P/AP+

84 CHR-P/AP-

T2

*153* CHR-P individuals

*concluded* the 2-year follow-up period

*22* CHR-P individuals

*service disengagement*

83 CHR-P/AP+

70 CHR-P/AP-

*6* CHR-P individuals

*dropped-out* from the PARMS program

*12* CHR-P individuals

conclusion for *clinical improvement*

*4* CHR-P individuals

*outside the catchment area*

Note. AP = Antipsychotic medication; CHR-P = Clinical High Risk for Psychosis; CHR-P/AP+ = CHR-P individuals with baseline AP prescription; CHR-P/AP- = CHR-P individuals AP-naive; T0 = baseline assessment time; T1 = 1-year assessment time; T2 = 2-year assessment time; PARMS program = Parma At-Risk Mental States.

Table S2 – Baseline sociodemographic and clinical comparisons between the two CHR-P subgroups (n = 180).

| Variable | CHR-P/AP+  (n = 92) | CHR/AP-  (n = 88) | X^2^/z | p |
| --- | --- | --- | --- | --- |
| Gender (males)  Ethnic group (white Caucasian)  Migrant Status  Age (at entry)  Education (in years)  *Civil status*  Single  Married/partnership  *Living status*  Alone  Living with partners  Living with parents  *Occupation*  Unemployed  Employed  Student  *Source of referral*  Primary care  Family members  Self-referral  Emergency room  School/Social services  Other mental health services  DUI (in weeks)  Previous hospitalization  Previous suicide attempts  Previous specialist contact  Substance misuse at entry  First-degree relative with psychotic disorder  *Baseline PANSS scores*  Positive Symptoms  Negative Symptoms  Disorganization  Affect  Resistance/Excitement  PANSS total score  Baseline GAF score | 45 (48.9%)  80 (87.0%)  14 (15.2%)  20.83±3.37  11.58±2.63  89 (96.7%)  3 (3.3%)  3 (3.2%)  3 (3.2%)  86 (93.6%)  35 (38.0%)  6 (6.6%)  51 (55.4%)  37 (40.2%)  6 (6.5%)  9 (9.8%)  26 (28.3%)  5 (5.4%)  9 (9.8%)  41.32±42.63  21 (22.8%)  8 (8.7%)  44 (47.8%)  18 (19.6%)  32 (34.8%)  11.40±3.90  19.79±6.75  16.19±5.50  16.07±5.24  6.89±2.76  72.81±17.15  47.67±7.16 | 45 (51.1%)  79 (89.2%)  14 (15.9%)  18.17±3.74  11.06±2.21  87 (98.9%)  1 (1.1%)  1 (1.1%)  2 (2.3%)  85 (96.6%)  21 (23.9%)  4 (4.5%)  63 (71.6%)  41 (46.6%)  9 (10.2%)  7 (8.0%)  15 (17.0%)  8 (9.1%)  8 (9.1%)  51.67±54.26  7 (8.0%)  11 (12.5%)  39 (44.3%)  13 (14.8%)  27 (30.7%)  10.18±3.59  17.97±7.43  15.18±5.20  14.78±5.11  7.57±3.55  68.77±17.51  49.83±9.82 | .089  .346  .016  -4.722  -1.659  .470  .976  3.469  4.671  -.984  7.573  .689  .223  .725  .343  -2.123  -2.531  -1.037  -1.301  -.659  -1.265  -2.198 | .766  .556  .898  **.0001**  .097  .601  -  -  .807  -  -  -  .263  -  -  -  .457  -  -  -  -  -  -  .325  **.006**  .406  .673  .395  .558  **.045**  **.048**  .300  .193  .510  .206  **.043** |

Note. CHR-P = Clinical High Risk for Psychosis; AP = Antipsychotic medication; CHR-P/AP+ = CHR-P individuals with baseline AP exposure; CHR-P/AP- = CHR-P individuals without baseline AP prescription; DUI = Duration of Untreated Illness; AP = Antipsychotic medication; AD = Antidepressant medication; MS = Mood Stabilizer; BDZ = Benzodiazepine; BLIPS = Brief Limited Intermittent Psychotic Symptoms; APS = Attenuated Psychotic Symptoms; GV = Genetic Vulnerability; DSM-5 = Diagnostic and Statistical Manual of mental disorders - 5^th^ Edition; NOS = Not Otherwise Specified; PANSS = Positive And Negative Syndrome Scale; GAF = Global Assessment of Functioning. Frequencies (and percentages) and mean ± standard deviation are reported. Chi-square (X^2^) test and Mann-Whitney U test (z) values are reported. Statistically significant p values are in bold.

DUI = Duration of Untreated Illness (defined as the time interval between the onset of psychiatric symptoms and the first pharmacological and/or psychological treatment) (Matsumoto et al., 2021). Suicide attempt = potentially injurious, self-inflicted behavior without a fatal outcome for which there was (implicit or explicit) evidence of intent to die (Silverman et al., 2007), derived from direct information reported by the patient (or by a relative well informed about the facts) or documented in the clinical notes.

Figure S2 – Profile plots for mixed-design ANOVA results between the two CHR-P subgroups (n = 180).


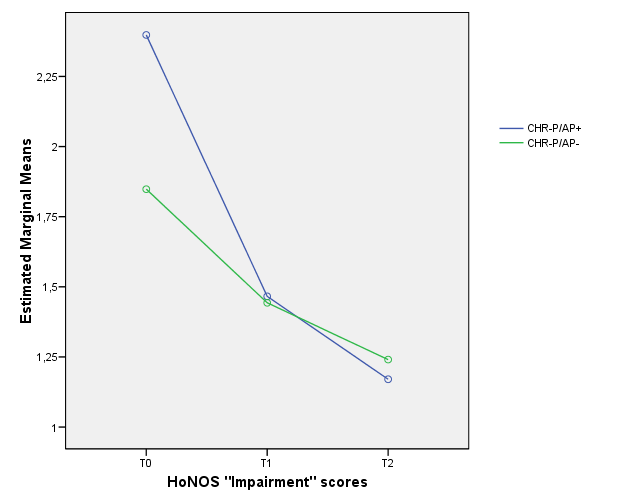

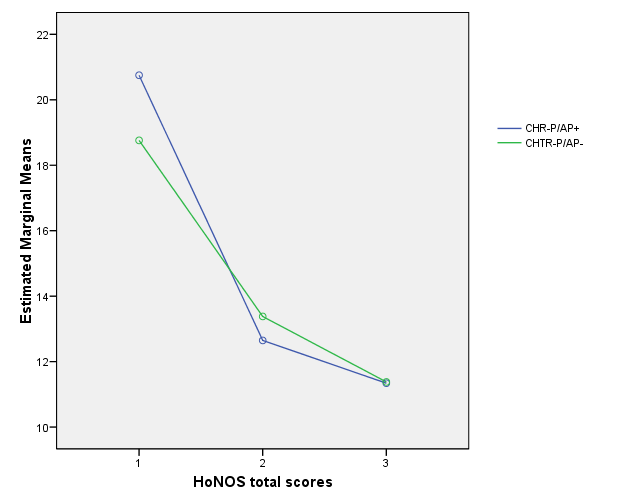


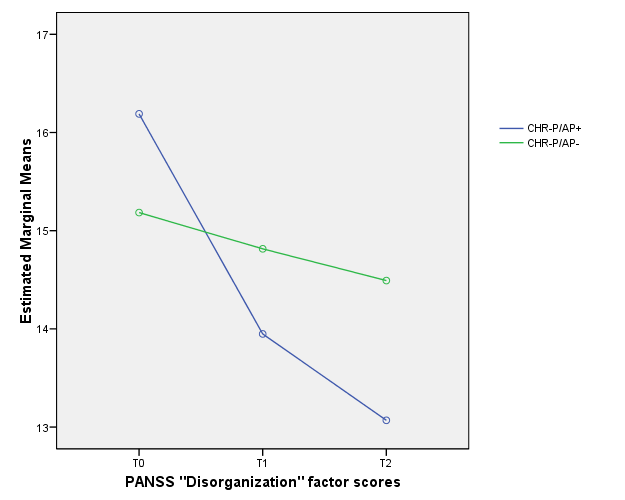


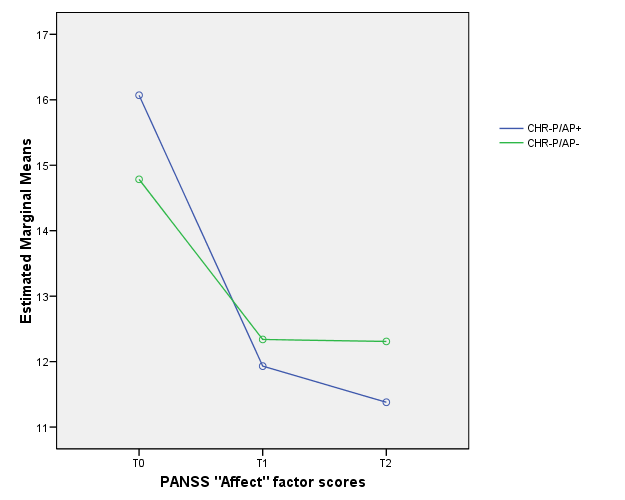


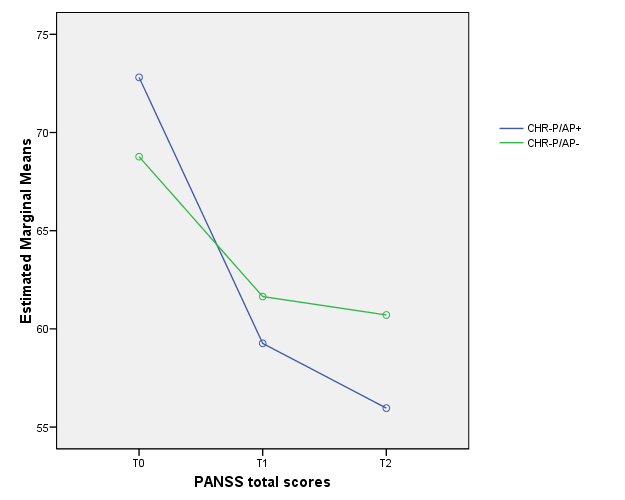


Note – ANOVA = Analysis of Variance, CHR-P = Clinical High Risk for Psychosis; AP = Antipsychotic; CHR-P/AP+ = CHR-P individuals with AP prescription at baseline; CHR-P/AP- = CHR-P individuals without AP prescription at baseline; HoNOS = Health of the Nation Outcome Scale; PANSS = Positive And Negative Syndrome Scale; T0 = baseline assessment time; T1 = 1-year assessment time; T2 = 2-year assessment time.
